# Aldehydes and ketones in pyrolysis oil: analytical determination and their role in the aging process

**DOI:** 10.1039/d1ra08899h

**Published:** 2022-03-04

**Authors:** Clarissa Baehr, Gavin J. Smith, Daniel Sleeman, Thomas A. Zevaco, Klaus Raffelt, Nicolaus Dahmen

**Affiliations:** Institute of Catalysis Research and Technology (IKFT) Hermann-von-Helmholtz-Platz 1 76344 Eggenstein-Leopoldshafen Germany Klaus.Raffelt@kit.edu

## Abstract

Aldehydes and ketones are known to play a role in the aging process of pyrolysis oil and generally, aldehydes are known for their high reactivity. In order to discern in pyrolysis oil the total aldehyde concentration from that of the ketones, a procedure for the quantification of aldehydes by ^1^H-NMR was developed. Its capability is demonstrated with a hardwood pyrolysis oil at different stages of the aging process. It was treated by the Accelerated Aging Test at 80 °C for durations of up to 48 h. The aldehyde concentration was complemented by the total concentration of carbonyls, quantified by carbonyl titration. The measurements show, that the examined hardwood pyrolysis oil contained 0.31–0.40 mmol g^−1^ aldehydes and 4.36–4.45 mmol g^−1^ ketones. During the first 24 h, the aldehyde concentration declined by 23–39% and the ketone concentration by 9%. The rate of decline of aldehyde concentration slows down within 24 h but is still measureable. In contrast, the total carbonyl content does not change significantly after an initial decline within the first 4 h. Changes for vinylic, acetalic, phenolic and hydroxyl protons and for protons in the *α*-position to hydroxy, ether, acetalic and ester groups were detected, by ^1^H-NMR. In the context of characterizing pyrolysis oil and monitoring the aging process, ^1^H-NMR is a reliable tool to assess the total concentration of aldehydes. It confirms the reactivity of aldehydes and ketones and indicates their contribution to the instability of pyrolysis oil.

## Abstract 2

A chemical-analytical procedure by ^1^H-NMR was developed to determine the total concentration of aldehydes in a hardwood-based pyrolysis oil during the process of accelerated aging at 80 °C for 48 h. It is compared to results of carbonyl titration.

## Graphical abstract

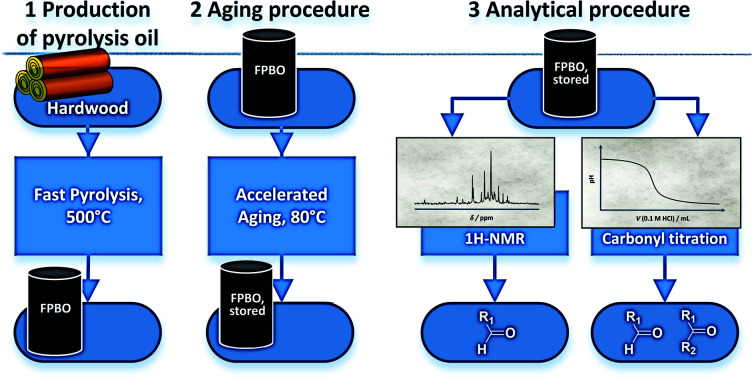

## Introduction

Traditionally, biomass has been extracted for diverse purposes. Besides nutritional reasons, it has always served as a material in the construction sector^1^ for instance and as a common direct source of energy.^2^ The long history of biomass utilization and its commonplace nature demonstrate how biomass is a vital, but publicly often underrecognized commodity. However, its way of utilization can be an important factor in the necessary changes of today's supply chain arising from earth's limited resources and ongoing climate change.^3–7^ Biomass binds carbon dioxide and can act as a renewable resource including the production of raw chemicals and energy carriers.^8–11^ Because this kind of biomass utilization should not impede or compete with food production, ways to use agricultural waste material are of growing interest.^9,11,12^

One technology, which has the potential to convert biomass within these parameters is the fast pyrolysis process.^13^ It breaks down biological macromolecules like lignin, hemicellulose and cellulose into smaller molecules^14^ and yields high amounts of liquid products when compared to other thermochemical conversion processes. It is characterized by a short residence time of the vapour of *ca.* 1s, a reactor temperature of 500 °C, ambient pressure and the exclusion of oxygen. It is ended by a rapid condensation of the reaction mixture, which leads to the liquid product and an incomplete degradation of the biomass.^15^ The gained liquid product fraction is generally referred to as fast pyrolysis bio oil (FPBO), which is a promising intermediate carrier for renewable energy.^13^ Its composition depends strongly on the process parameters and the properties of the used biomass.^16^

So far, the commercial application of pyrolysis oil is the usage as fuel for industrial burners. This is reflected by the recommended application in the norms EN 16900:2017 ^17^ and the ASTM D7544-10.^18,19^ Commercial plants are operated, *inter alia*, by Twence/Empyro BV (Netherlands),^20^ Fortum (Finland)^19^ and Envergent Technologies' RTP™ (Canada),^21^ who use wood-based biomass. The application as a burner fuel requires a defined viscosity and a reliable storage time in order to transfer pyrolysis oil by pumping to the burner and to ensure good atomization.^15,18^ Therefore, the ASTM D7544-10 ^18^ also defines that pyrolysis oil shall stay uniform for at least 3 months of storage (medium-term storage). Research on other applications, in for example diesel-engines, gas-turbines or its use as a feedstock for chemicals, are still underway.^19,22^
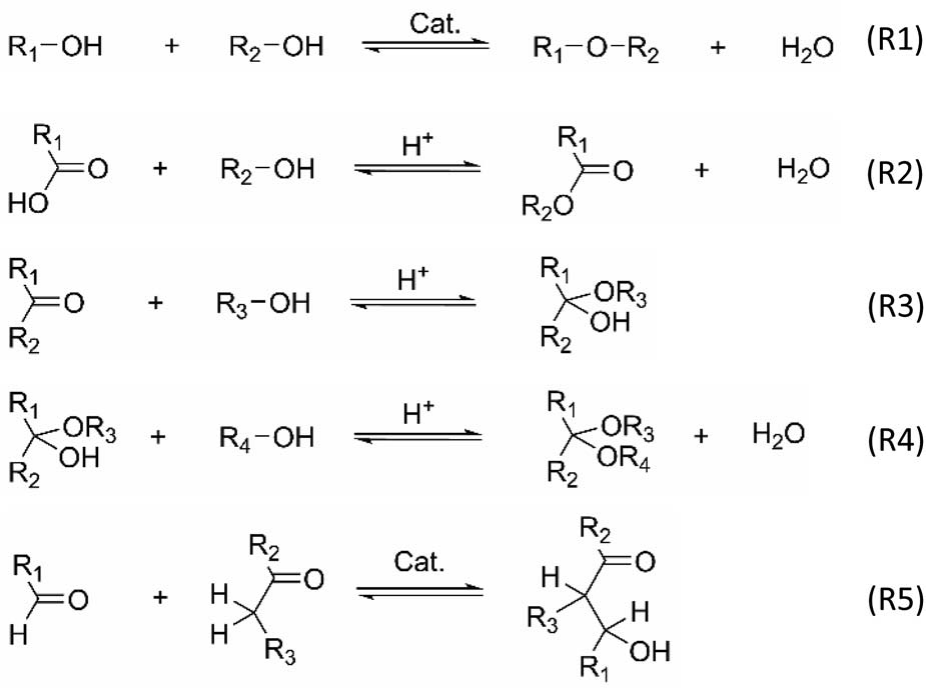


The application of pyrolysis oil is limited because of its difference to mineral oil regarding composition and properties.^19^ Pyrolysis oil is composed of more than 300 substances, including water, high-molecular substances like pyrolytic lignin, phenols and many oxygenated compounds like sugars, alcohols, aldehydes, ketones, ethers and organic acids.^23–27^ Their composition determines their properties and their tendency to change during storage (aging). Particularly, the high water content reduces the heating value,^15^ the fraction of high-molecular components causes their high viscosity^28^ and the presence of acidic compounds leads to a low pH value.^19^

Aging is mainly observed by an increase in viscosity and possible phase separation. It is accompanied by an increase in molar mass, an increase of water content and a significant decrease of compounds displaying carbonyl groups.^29–31^ The increase of the average molar mass correlates with the aging of the samples, manifesting as a viscosity increase and a possible phase separation.^32^ These issues are all indicators of the stability (thermal and chemical) of the pyrolysis oil and its storage capability.^33–36^

The extent of aging depends on the specific pyrolysis oil, the storage time and the temperature. The higher the storage temperature the faster this process proceeds. To compare the aging behaviour of different pyrolysis oils a procedure called Accelerated Aging Test was developed by Oasmaa and Elliott *et al.* In this case pyrolysis oil is stored for 24 h at 80 °C in sealed glass bottles.^29,37^

The underlying cause of aging is thought to originate from reactions involving reactive compounds in pyrolysis oil, that lead to larger molecules with changed polarity.^18,33,38,39^ In this context, several reactions have been proposed as a potential cause of aging, among them etherification (R1), esterification (R2) hemi-/acetalization (R3, R4), aldolization (R5), redox reactions of alcohols, and several polymerizations.^29,33,36,40–45^ In regard to these frequently mentioned aging reactions, carbonyls show a high reactivity, of which aldehydes are the most reactive. Interestingly, purposeful esterification and hemi-/acetalization with low-molecular, mono-functional alcohols are also exploited to lower the aging reactions and thus stabilize pyrolysis oil.^33,46–48^

These reaction types have been identified by monitoring the concentration changes of numerous key components present in the pyrolysis oil, during the aging process. Analytical techniques include mainly Karl-Fischer titration, Fourier-transform infrared spectroscopy (FTIR), nuclear magnetic resonance spectroscopy (NMR), carbonyl titration and gas chromatography-mass spectrometry (GC-MS).^29,30,33,40,43,44,49–52^

Karl-Fischer titration determines the amount of water in pyrolysis oil. During aging at 80 °C or 90 °C respectively for 24–30 h a slight increase of water in pyrolysis oil can be observed, amounting to *ca.* 0.5–2 mmol g^−1^.^37–40,53^ Complementary, non-destructive, spectroscopic methods can determine the organic compounds present in pyrolysis oil and shed light on the complex product ranges.

FTIR measurements have revealed that compounds with C–O–H, C–O–C and C=O bonds are present in pyrolysis oil, probably belonging to alcohols, esters, ethers, acetals and carbonyls. During accelerated aging experiments, it has been qualitatively suggested, that the concentration of esters increases. Similarly FTIR data also suggests an increase of carbonyl concentration over time. This is not consistent with findings gained from carbonyl tritration.^38,40,43,44^ Carbonyl titration shows a clear decrease of up to 30–50% of carbonyl compounds within 24 h during accelerated aging, but without distinguishing between different carbonyl groups such as aldehydes and ketones.^29,30^

An approach using ^13^C-NMR has confirmed the tendency of decreasing concentration during aging, albeit with low precision. Corresponding ^1^H-NMR measurements for aldehydes showed no significant change in concentration.^50^ Approaches using ^13^C- and ^19^F-NMR can be seen as more elaborate.^50,52^ The ^13^C-NMR is somewhat limited due to its low sensitivity (natural abundance 1.1% against 100% for ^1^H) and the difficulty to quantitatively assess the different species present in complex mixtures. This might explain the sparse reports about quantitative characterization using ^13^C-NMR-techniques. On the other hand ^1^H-NMR has been revealed to be more practical: in this case aldehydes can be easily quantified because of their unique chemical shift^54,55^ and contrary to other substance classes one proton can be assigned to each aldehyde group. In the chemical shift range characteristic for aldehydes, there are no major interferences with other substance classes present in the pyrolysis oil. Furthermore, the sample preparation is simple and only small amounts of pyrolysis oil are necessary due to the higher sensitivity of ^1^H- compared to ^13^C-NMR spectroscopy.

In comparison to the previous analytical methods, GC-MS can only characterize parts of pyrolysis oil. It is suitable to measure substances of low to medium weight, but none belonging to the wide range of oligomeric pyrolytic lignin, even though, these amount to a third of the total mass of pyrolysis oil. Apart from that, quantification by GC-MS requires a calibration, meaning that many substances have to be known beforehand to be clearly identified and quantified. Consequently, it is rather complicated to unambiguously quantify any given compound class in pyrolysis oil using GC-MS.^25,56^

In our approach ^1^H-NMR is used to determine aldehydes in pyrolysis oil with a high precision, allowing monitoring the aging process of a hardwood pyrolysis oil. The pyrolysis oil was aged at 80 °C for up to 48 h. NMR measurements were conducted with two different spectrometers using frequencies of 400 MHz and 44 MHz for ^1^H-NMR. Combining ^1^H-NMR data with results from carbonyl titration the ketone concentration can be additionally assessed. By doing this, the pyrolysis oil and its aging process can be described by the aldehyde and ketone concentration, which can be seen as key-indicators in this context.

Additionally, the ^1^H-NMR spectra were used to draw more information on other compound classes, *e.g.* alkanes, aromatics, vinyls and phenols. Ultimately, this data will be discussed in the context of aging reactions, considering not only concentration changes, but also specific reaction conditions in pyrolysis oil. Hereby, the analytical results of this work will be discussed together with literature-known correlations gained from FTIR spectra.

## Experimental

The pyrolysis oil was produced with the hardwood product LIGNOCEL (J. RETTENMAIER & SÖHNE GMBH + CO KG) using the process development unit ‘Python’ of the bioliq®-process operated at the Institute of Catalysis Research and Technology at Karlsruhe Institute of Technology, that is described elsewhere.^57,58^ The pyrolysis took place at 500 °C and the condensation at 90 °C. The feed rate was at 7 kg h^−1^.

The whole pyrolysis oil was aged based on the accelerated aging procedure of Oasmaa and Elliott *et al.*^29,37^ Samples with 7.0 g whole pyrolysis oil in sealed vials with screw top (Supelco, 20 mL) were stored at 80 °C for up to 48 °h in an oven unit for gas chromatography (Hewlett Packard 5890 Series II Plus). Sampling was performed in time intervals of 1, 2, 4, 8, 12, 24 and 48 h.

For each time interval, three samples were prepared. Thus, the aging procedure was repeated three times and consecutively, each sample was analyzed by ^1^H-NMR and carbonyl titration. The original pyrolysis oil was analysed in the same manner, including the determination of the water content by Karl-Fischer titration.

The carbonyl titration followed the method of Black/Ferrell.^30,31^ Karl-Fischer titration was performed by a Titrando 841, Metrohm. ^1^H-NMR spectra were recorded using a Varian InovaUnity spectrometer with an 9.4T Oxford cryomagnet (^1^H frequency at 399.9 MHz; 128 scans for each samples, pulse width 6.35 μs, spectral width 6400 Hz, time domain 16k) and a benchtop spectrometer Magritek Spinsolve with a permanent 1T Halbach array Magnet (^1^H frequency at 43.76 MHz; 40 scans for each samples, pulse width 9.60 μs, spectral width 5000 Hz, time domain 33k). ^1^H-NMR samples contained 50 mg of pyrolysis oil dissolved in 800 mg of DMSO solution, that contained 1 mg 3-(trimethylsilyl)propionic-2,2,3,3-*d*_4_ acid sodium salt (a.k.a. TMSP, Alfa Aesar, >98% D) per 1 mL DMSO-*d*_6_ (Sigma-Aldrich, 99.9% D). Before transfer into NMR-tubes, the samples were centrifuged to remove solids. The substances used as reference were benzaldehyde (Merck, extra pure), syringaldehyde (Alfa Aesar, >98%), vanillin (Merck, >99%), propionaldehyde (Fluka, 98%), furfural (Merck, 98%), coniferyl aldehyde (Sigma-Aldrich, 98%), acetoin (95%) and ethyl acetate (Merck, 99.5%). Phase and baseline corrections of the spectra, as well as any further spectrum processing (*e.g.* the integration of the signals) were done manually with MestreNova (version 14.2.0-26256) like any further spectrum processing, *e.g.* the integration of the signals. The internal standard (trimethylsilyl)propionic-2,2,3,3-*d*_4_ acid (TMSP-*d*_4_) was used to calibrate the spectra (0 ppm) and for further quantification. The integration ranges are shown in Table 1.

**Table 1:** ^1^H NMR chemical shift assignment of functional groups^59^

*δ* range/ppm	Proton assignments
−0.05–0.05	Internal standard TMSP-*d*_4_
1.5–0.5	Alkyl groups
3.0–1.5	Alkyl groups in α-position to carbonyl and carboxyl groups
4.3–3.0	Alkyl groups in α-position to alcohol, ether and ester groups, alkoxy groups of acetals
6.0–4.3	Vinylic moieties, acetals (protons in α-position to acetalic alkoxy groups), hydroxy groups of alcohols and phenols
8.5–6.0	Aromatics, hydroxy groups of alcohols and phenols
10.4–9.2	Aldehydes

## Results & discussion

### ^1^H-NMR

^1^H-NMR spectra of pyrolysis oil give direct information on its chemical composition with a focus on specific functional groups and on their direct surrounding in correlation with their ^1^H chemical shift. Spectral analysis thus allows some inferences on the distribution of compound classes present in pyrolysis oil.

**Fig. 1:**
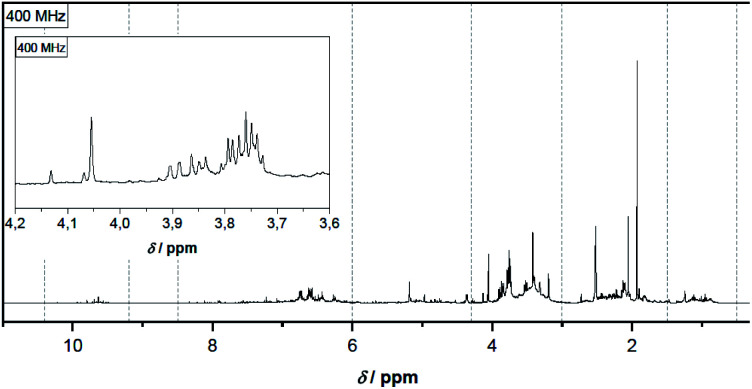
^1^H-NMR spectrum of hardwood pyrolysis oil before aging.

This work focuses on molecules containing alkyl groups in the proximity of electron-withdrawing groups like *e.g.* vinylic, acetalic and aromatic moieties, and particularly aldehyde groups. These ^1^H signals can be seen as shifted to lower fields (deshielded and at higher chemical shift values) and hence easily distinguishable from the bulk of the remaining ^1^H alkane signals. As it can be seen in Fig. 1 the chemical shift regions in the spectrum are very different in intensity, clearly indicating different concentrations of compounds. Protons of alkyl groups in α-position to electron-withdrawing groups such as carbonyl, carboxyl, hydroxy, ether and ester groups are particularly distinctive. Whereas alkyl groups not in α-position to those groups cannot be clearly distinguished due to multiple overlaps. The highest signal, that could be attributed to a methyl group, namely that of acetic acid, is at 1.93 ppm, and the solvent signal appears at 2.52 ppm. Reference data^60^ lists the methyl group of acetic acid in the same range at 1.91 ppm in DMSO-*d*_6_ whereas the solvent signal is at 2.50 ppm. Adding a small amount of water results in a characteristic signal at 4.06 ppm.

Analogously, the chemical shift for several aldehydic protons was determined. The results are summarized in Table 2. Vanillin and syringaldehyde have a similar chemical shift of 9.79–9.80 ppm, due to their structural similarity. Therefore, it may be assumed that similar aldehydes might have overlapping signals in this range, as well. Other assigned aldehydic protons belong to benzaldehyde, propionaldehyde, furfural and coniferyl aldehyde.

**Table 2:** ^1^H-NMR chemical shift assignment of some aldehydes

Substance	No.	Structure	*δ*/ppm
Benzaldehyde	—	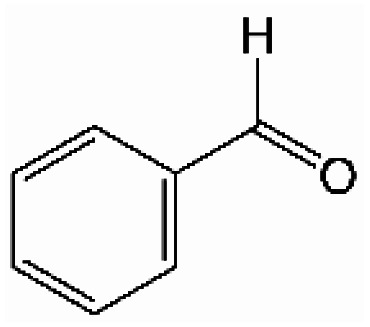	10.03
Syringaldehyde	1	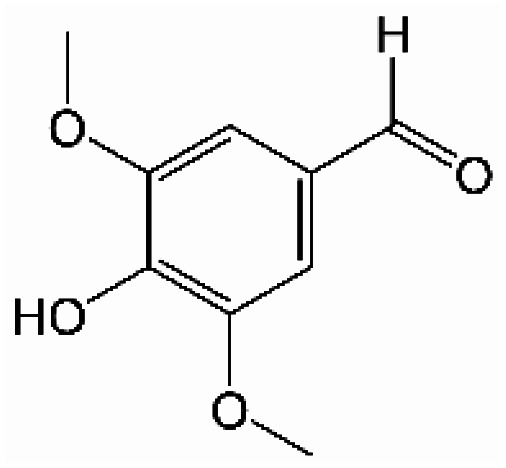	9.80
Vanillin	2	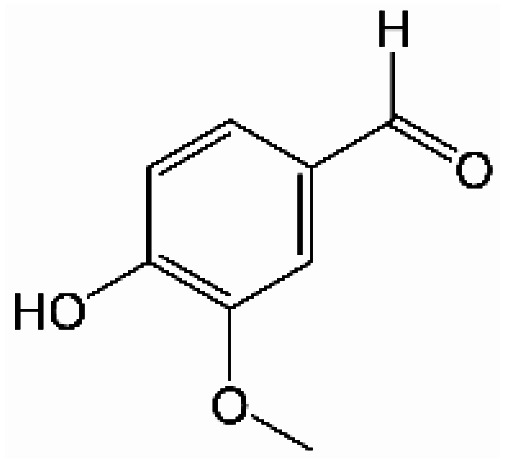	9.79
Propionaldehyde	3	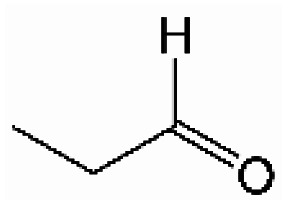	9.70
Furfural	4	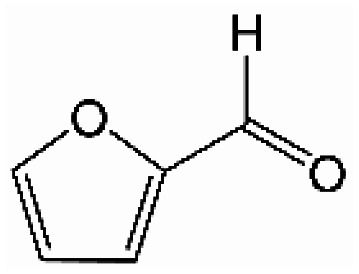	9.65
Coniferyl aldehyde	5	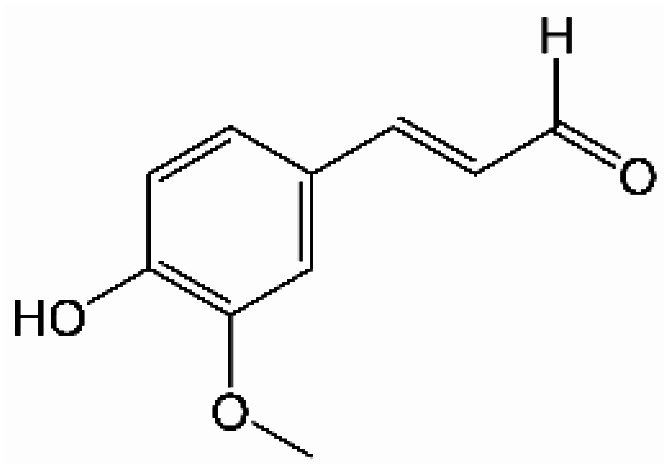	9.62; 9.60

**Fig. 2:**
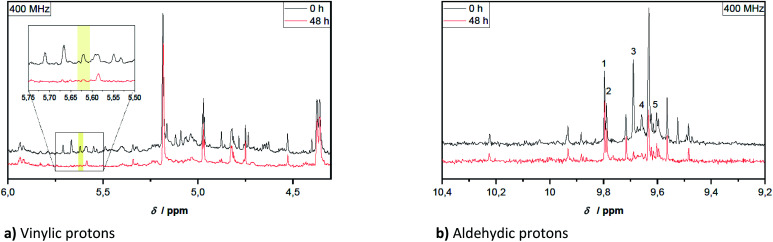
Sections taken from ^1^H-NMR spectra of hardwood pyrolysis oil before and after 48 h aging at 80 °C. Sections are differently scaled for enhanced comparison.

In Fig. 2 two spectra are compared, subdivided into two characteristic regions, before and after aging of the pyrolysis oil (Fig. 2). The aging was performed at 80 °C for 48 h.

Qualitatively noticeable is the lower intensity of many signals in the spectrum of the aged pyrolysis oil and the disappearance of some characteristic signals. This phenomenon was also observed for the vinylic structures and partly for protons in α-position to the hydroxy, ether and ester groups. The aromatic structures changed less during aging. The clearly visible signal at 3.79 ppm (quartet) in Fig. 1 can be tentatively assigned to an ethyl group next to a strong electron-withdrawing group. Even though, acetoin and ethyl acetate are plausible assignments, this could be excluded by specific sample spiking (controlled addition to a pure sample to confirm a signal attribution).

For the aldehydic region in Fig. 2b several signals can be assigned to known aldehydes as summarized in Table 2. The exception to this is benzaldehyde, which was not be detected in this specific pyrolysis oil. Signals tentatively assigned to syringaldehyde, propionaldehyde and furfural, diminish during the aging procedure. It is noteworthy, that the chemical shifts of individual compounds are dependant on the accompanying substances included in the overall mixture. This is why, individual chemical shifts can be specific to a pyrolysis oil.

**Fig. 3:**
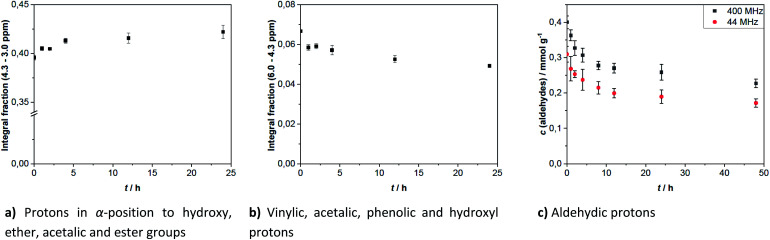
Several ^1^H-NMR integration results for hardwood pyrolysis oil with aging time, in case of aldehydes converted to concentration.

Generally, the aging process can be monitored by ^1^H-NMR, because it allows to follow the change of hydrogen bonds during chemical reactions. The integration ranges for each signal type are defined in Table 1. These integration values are used to calculate the results in Fig. 3. They are given as the area fraction, which is defined as the ratio of the specific integration area of a defined range to the integration area of the total measuring range. The area fraction for protons in α-position to simple O-bonded groups increased and the area fraction of vinylic, aromatic and aldehydic protons decreased. The increase of area fraction for protons close to simple O-bonded groups (hydroxyl, ether-, acetal- or ester-groups) indicates the increase of at least one of these groups. This points towards esterification and hemi-/acetalization as aging pathways, that are often discussed in the literature.^29,33,36,40,41,44,45,61^ The increase of the area in the range of 4.3–3.0 ppm cannot be explained by the water signal, because no clear change in the intensity of the signal was identified. This is probably due to the comparatively low amount of water generated by aging reactions.

Hemi-/acetalization can be indicated by signals in two ranges. The alkoxy group can be identified in the region of the ethers (see Table 1). Protons adjacent to acetal groups are more in the range around 5.5 ppm.^62,63^ In this range, a signal at 5.59 ppm can be clearly seen after aging of the samples. This was tentatively assigned to hemi/-acetals, indicating that acetalization has occurred.

Vinylic compounds are known to be reactive and to easily undergo electrophilic addition and [2 + 4] cycloadditions. In pyrolysis oil they were detected in degradation products of lignin like canolol, isoeugenol and coniferyl alcohol. Pericyclic reactions, [2 + 4] cycloadditions a.o., can lead to molecules with significantly higher molar mass. From the literature dealing with gel permeation chromatography (GPC) it is known, that the average molecular mass increases significantly during the aging of pyrolysis oil. This is also evident for fractions, that can be separated by filtration or extraction.^38–41,53,64^ In addition, there is a clear correlation between the increase in mean molar mass and enhancement of the viscosity.^29^

For the aldehydic protons, the integration area of the corresponding region (10.4–9.2 ppm) can be related to the concentration of aldehydes in pyrolysis oil by means of the internal standard TMSP. This is because aldehydes possess exactly one aldehydic proton per aldehyde group, contrary to the other compound classes. The concentration of aldehydes at the beginning was 0.31–0.40 mmol g^−1^. As depicted in Fig. 2b, some aldehyde signals disappear completely over the time, like these of propionaldehyde (3) and furfural (4). Signals of other aldehydes instead are quite constant during aging, belonging clearly to the more stable aldehydes. Single values for the aldehyde concentration during aging are given in Table 3. The relative standard deviation of the measurements in triplicate corresponds to 4–9% for the aldehyde concentration determined at 400 MHz and 4–13% for measurements at 44 MHz.

**Table 3:** Aldehyde, ketone and carbonyl concentration in hardwood pyrolysis oil with aging time, the higher aldehyde concentrations belong to NMR measurements at 400 MHz and lower to measurements at 44 MHz

*t*/h	*c* (aldehydes)/mmol g^−1^	*c* (ketones)/mmol g^−1^	*c* (ketones)/*c* (aldehydes)	*c* (carbonyls)/mmol g^−1^
0	0.31–0.40	4.36–4.45	11–14	4.76
4	0.24–0.31	3.89–3.98	13–17	4.21
24	0.19–0.26	4.00–4.07	15–21	4.26

As with the vinylic protons, no stable aldehyde concentration was reached within 48 h. Although, the aldehydes seem to be more reactive, due to their steep decline during the first hours. Most aldehydes reacted during the first 10 h of aging. After 24 h 61–77% aldehydes are left. The remaining seem to be less reactive as the decrease in concentration has significantly slowed. The analysis of spectra recorded at 44 MHz lead to lower concentrations, probably due to the lower resolution of the spectra, impeding the detection of weaker signals.

In acidic media, aldehydes can react by hydration and hemi-/acetalization, which are probably concurrent reactions in pyrolysis oil. For hydration the equilibrium constant is around 1 for most aldehydes in pyrolysis oil.^65^ Thus, depending on the water content of the pyrolysis oil, hydration can reduce the aldehyde amount that is available for other reactions.

Yields for hemi-/acetalizations are less clear. Acetal formations and acetal hydrolyses conducted without a solvent in the presence of a heterogeneous catalyst have varying yields. For the hydrolysis of benzaldehyde dimethyl acetal the equilibrium constant is *ca.* 10, with a shift to methanol and benzaldehyde.^66^ Acetalization of glycerol with benzaldehyde, *n*-heptaldehyde, *p*-anisaldehyde, furfural and acetone lead to conversions of 17–75%. In the case of smaller aldehydes higher conversions could be attained.^67,68^ Glycerol can lead to cyclic acetals that are entropically favoured.^65^ In comparison acetalizations occurring in pyrolysis oil have probably conversions below 75%, due to the complexity of the matrix and the less than optimal conditions. The aldehydes in pyrolysis oil possessing a high molar mass display significantly lower concentrations than in the referred experiments^66–68^ where specific catalysts were used. Additionally, the water content in pyrolysis oil might reduce the available aldehydes due to hydration. These processes can explain, why only around a third of aldehydes reacts during the aging procedure.

Sugars, more particularly oligosaccharides, can be a crucial component in the aging process, because they can take part in different ways in hemi-/acetalization reactions. Their versatile oligomeric structure, displaying polyhydroxy fragments together with aldehydes or ketones (resp. aldose or ketose as monomer) can lead to the formation of either glycosides or longer polysaccharides, which can both increase the viscosity.

### Carbonyl titration

The method of carbonyl titration can be used to measure the total amount of carbonyl compounds. Single values for the carbonyl concentration are listed in Table 3. The relative standard deviation of the triplicate samples corresponds to 1–3%.

**Fig. 4:**
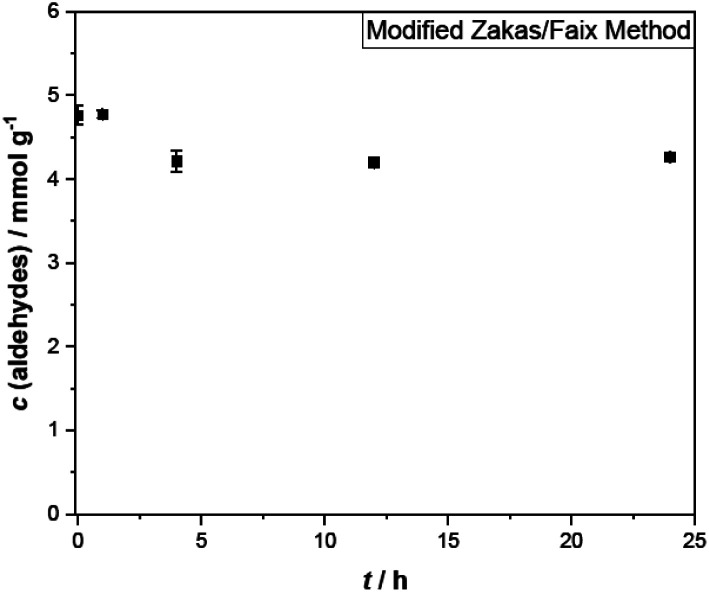
Carbonyl concentration in hardwood pyrolysis oil with aging time.

The determined total carbonyl concentration is an order of magnitude higher than the concentration of aldehydes alone (Fig. 4). Generally, studies on pyrolysis oil deploying carbonyl titrations report a carbonyl concentration between 4–6 mmol g^−1^.^29,30^ For the pyrolysis oil at hand, the starting carbonyl concentration is 4.8 mmol g^−1^. Aging diminishes the concentration to 4.3 mmol g^−1^ within 24 hours, which is comparable to results of other accelerated aging tests.^29,30^

Using the aldehyde concentration, the ketone concentration in pyrolysis oil can be deduced. Before the aging procedure the pyrolysis oil contained 4.4–4.5 mmol g^−1^ ketones. The error for the ketone concentration is estimated by the sum of the standard deviation of the aldehyde and ketone determinations and therefore, it is in the same range as the carbonyl titration.

After a 24 h aging period, the pyrolysis oil contained 4.0–4.1 mmol g^−1^ ketones. That corresponds to a reduction of 9%. Even though, ketones present in pyrolysis oil are less susceptible to further reactions than aldehydes, their higher initial concentration actually leads to a higher percentage share of reactions during the aging of pyrolysis oil.

These findings confirm the common knowledge that ketones are less reactive than aldehydes but also indicate that ketones are still important for describing the aging process. Ketones tend to follow similar reaction pathways to aldehydes, with hydration, hemi-/actelization and [2 + 4] cycloaddition as possible reaction pathways. However, these reactions proceed more slowly for ketones, because of steric and electronic reasons.^65,69^ For ketones in particular, hydration might only play a negligible role in comparison to the other two concurrent reaction pathways.^65^

Therefore, it is necessary to consider ketones, due to their absolute number involved in aging reactions, and aldehydes, because of their reactivity.

### The role of aldehydes and ketones in the aging process

The aging of pyrolysis oil is caused by a complex network of chemical reactions, even though aging is commonly described in the field by the physical parameter of viscosity increase. A chemical approach is difficult, because pyrolysis oil consists of a wide range of compounds with sometimes low concentrations, which make a thorough assignment and quantification of many of its compounds and compound classes difficult.

However, monitoring reactive compound classes *via* NMR can be a promising approach to assess the aging behavior of pyrolysis oil. Improved monitoring of the aging process can help in managing the stability issues of pyrolysis oil using the NMR data, general reaction pathways for aging can be proposed and the understanding of the aging reactions can be improved. Aldehydes and ketones play a key role owing to an important downstream chemistry with sugars, pyrolytic lignin and its degradation products. Possible reactions for ketones and aldehydes in pyrolysis oil are hydration, hemi-/acetalization and [2 + 4] cycloadditions. The first two reactions are influenced by the presence of water, which makes the water content of the oil an additional, and possibly underestimated factor in the aging process, whereas it is often only seen as a result of the aging process.

Aldol reactions are unlikely to occur in pyrolysis oil, because aldol additions with two aldehydes are only slightly exothermic and -additions with two ketones even slightly endothermic. They proceed mainly in the presence of basic catalysts.^65^ Therefore, they only proceed spontaneously in the case of aldehydes, if a suitable catalyst is present. Acid-catalyzed aldol reactions are known, but require very acidic conditions such as a saturation of the reaction mixture with hydrochloric acid.^70–73^ These conditions are not fulfilled in pyrolysis oil.

Several examples from the literature indicate that in addition to reactions involving alcohols and carbonyls, straightforward reactions involving carboxylic acids should also be considered.^40,43,44,74^ For instance, esterification is strongly suggested in FTIR spectra of pyrolysis oil^40,43,44,74^ by absorption bands at 1700 cm^−1^ and 1200 cm^−1^.^59^ Absorption around 1200 cm^−1^ can be assigned to many other compound classes like ethers (*ν*(C–O)), acids and especially sulfates (*ν*(S=O); *ν*_3_ mode of SO_4_ group), but signals around 1700 cm^−1^ usually belong to carbonyls, and coupled with the absorption band at 1200 cm^−1^ to esters (asymmetric and symmetric stretching modes of the carboxylic acid, *ν*_as_(COO^−^) and *ν*_s_(COO^−^) resp.).^59^ Nitrogen containing compounds are not considered here, because they are no common components in lignocellulose-based pyrolysis oil.^26^

Furthermore esterification is likely to happen, owing to the supplementary fact that it is catalysed in acidic media^54^ and organic acids are abundant in pyrolysis oil.^75^ Etherification is also suggested in the literature as a possible aging reaction, owing to the presence of FTIR absorption bands around 1200 cm^−1^ and 1050 cm^−1^. These bands are not specific, but can relate to compound classes displaying C–O bonds in their structures (stretching mode, *ν*(C–O)). While etherification is suggested in literature, it is unlikely to happen considering the reaction conditions in pyrolysis oil during accelerated aging. Etherification only takes place in the presence of strong mineral acids or certain catalysts, which are not present during aging of pyrolysis oil. Generally speaking, one should keep in mind that the infrared spectroscopy, although delivering important information on the functional groups present in complex mixtures, does not allow an easy and straightforward quantification of the compound classes present in such mixtures as is the case for NMR spectroscopy.

Acetalization and esterification can explain the formation of water during aging determined by Karl-Fischer titration, but these reaction types are not an obvious reason for the viscosity increase observed during aging. A significant viscosity increase implies the linkage of many small molecules or the coupling of molecules with high molar mass. This means, that molecules engaged in aging reactions must have more than one functional group and probably belong to the pyrolytic lignin fraction.

Esterification will take place with the most available organic acids in pyrolysis oil, which are usually formic and acetic acid.^75^ Because these molecules have a low molecular mass and are mono-functional, they probably do not contribute to the viscosity increase. Hemi-/acetalization involves carbonyl and hydroxyl groups,^65^ which are present in pyrolytic lignin, its degradation products and carbohydrates.^25,41,43,49,76,77^ Those molecules tend to have a high molar mass and display functional groups that are able to undergo cross-linking reactions. Performing experimental work with fractions of pyrolysis oil differing in average molar mass, it has been suggested, that the viscosity increase depends on the molar mass of pyrolytic lignin oligomers and their concentration in pyrolysis oil.^32^

Therefore, in contrast to esterification, the reaction of hemi-/acetalization is likely to play a major role in the increase of the viscosity. Possibly, [2 + 4] cycloadditions can also contribute to the linkage of molecules, which offer one more reaction pathway that could increase viscosity, although [2 + 4] cycloadditions are more likely to happen with vinylic compounds.

The separation of solids present in pyrolysis oil can mitigate the aging process, if explicitly separated before the storage, as indicated by some aging experiments.^37,41,44,78,79^ This strongly suggests that some components of the separated fractions are potential educts or catalysts for aging reactions.

## Conclusions

In terms of aging stability, a hardwood pyrolysis oil can be described, with a reasonable precision, by its total concentration of aldehydes and ketones by combining ^1^H-NMR measurements with carbonyl titration. Consequently, ^1^H-NMR is a useful tool that can also determine slight changes during the aging of pyrolysis oil.

Before aging the aldehyde content amounts to a concentration of 0.40 mmol g^−1^, when deduced from ^1^H-NMR spectra recorded at 400 MHz, whereas spectra at 44 MHz delivered a concentration of 0.31 mmol g^−1^. The standard deviation lies at 4–9% for measurements at 400 MHz and is only slightly higher for 44 MHz with 4–13%, owing to the general good resolution of the ^1^H spectra in the aldehyde region.

The corresponding ketone concentration is at 4.36–4.45 mmol g^−1^, which was calculated by the results of carbonyl titration and ^1^H-NMR measurements. The standard deviation is around 1–3%. The accelerated aging at 80 °C decreased the aldehydes by 23–39% and ketones by 9% within 24 h.

Under the acidic conditions present in pyrolysis oil hemi-/acetalization reactions involving carbonyls and several components of the pyrolytic lignin fraction and its degradation products can be seen as the most probable aging reactions. ^1^H-NMR spectra indicate an increase in C–O bonds, that is possibly related to acetals and esters, which is in agreement with FTIR data from the literature.^40,43,44,74^

Pyrolytic lignin reacting in aging reactions can explain the increase of viscosity that is characteristic for the instability of pyrolysis oil. Because the information on high-molecular components is sparse, the knowledge of aging could be improved by regularly deploying chemical-analytical methods able to gain information on pyrolytic lignin, its degradation products and sugars, as well.

As a consequence, the aldehyde concentration can be a practical chemical parameter to assess the stability of pyrolysis oil and monitor the aging process. The determination of the concentration of aldehydes in pyrolysis oil using ^1^H-NMR nicely complements the information gained by carbonyl titration, especially in respect of the classification of carbonyls into aldehydes and ketones.

## Conflicts of interest

There are no conflicts to declare.

## Supplementary Material
